# Exploration and practice of Medical Simulation Center construction under the background of New Medical Sciences

**DOI:** 10.3389/fpubh.2025.1619348

**Published:** 2025-11-21

**Authors:** Aiying Zeng, Xueqing Chen, Quanmin Wu, Xiuhua Lin, Xiaorui Peng, Qinyong Ye

**Affiliations:** 1 Fujian Medical University Clinical Skills Teaching Center, Fuzhou, Fujian, China; 2 Fujian Medical University Union Hospital, Fuzhou, Fujian, China

**Keywords:** medical simulation, competency-based education, simulation-based learning, healthcare training, objective structured clinical examination

## Abstract

**Background:**

Medical Simulation Centers have become integral to filling the gaps between theoretical learning and clinical practice as part of modern medical education. Traditional clinical teaching is often restricted by ethical constraints, limited patient contacts, and variable quality of training; there is a need for structured, simulation-based approaches to fill those gaps.

**Objective:**

This study is intended to identify effective approaches to developing and optimizing Medical Simulation Centers based on the New Medical Sciences principles, and the Fujian Medical University institutional experience is proposed as a model.

**Methods:**

A descriptive institutional study was designed to collect data from Fujian Medical University’s Medical Simulation Center from 2020 to 2024. Key elements of infrastructure development, management components, faculty development, and curriculum development were considered, emphasizing principles of competency-based medical education.

**Results:**

Developing a structured simulation-based program improved students’ clinical preparedness, procedural skills, and performance in objective structured clinical examinations (OSCE). The Fujian model shows that positive results related to faculty development, sustainable funding, and curriculum integrations are important to the success of Medical Simulation Centers.

**Conclusion:**

Medical Simulation Centers are essential to bridging theory and practice in medical education. Creating a structured and governed simulation program improves educational quality, educators’ capacities, and sustainability, benefiting long-term education and clinical outcomes in health care.

## 1 Introduction

In recent years, the concept of New Medical Sciences has emerged as a transformative initiative in medical education, aiming to align healthcare training with the demands of modern medicine, scientific innovation, and interdisciplinary integration. Against this backdrop, Medical Simulation Centers have emerged as essential platforms for bridging the gap between theoretical education and practical clinical training. Traditional medical education often relies on real patient interactions, but with the increasing number of students, ethical concerns, and limited patient availability, it has become imperative to adopt alternative teaching methods. Simulation-based learning enables medical students to develop critical skills in a controlled environment, improving their confidence and clinical decision-making abilities before transitioning to real-world settings (1, 2). Additionally, competency-based medical education (CBME) frameworks increasingly integrate simulation-based training to provide structured, measurable learning experiences (3).

Despite its advantages, the establishment and operation of Medical Simulation Centers face several challenges. Many institutions struggle with inadequate funding, a lack of trained faculty, and inconsistent integration of simulation-based learning into the curriculum. Studies have shown that while some well-funded institutions possess state-of-the-art simulation facilities, these resources are often underutilized due to scheduling inefficiencies and insufficient faculty training (4, 5). Furthermore, disparities in Medical Simulation Center access across different medical schools raise concerns about the equitable distribution of high-quality clinical training (6). Addressing these challenges requires a strategic approach to Medical Simulation Center management, including investment in faculty development, resource allocation, and curriculum integration.

This manuscript explores the significance of Medical Simulation Centers and the challenges associated with their development. Drawing from the experience of Fujian Medical University, we propose strategies for optimizing Medical Simulation Center construction, including strengthening infrastructure, implementing effective management systems, and incorporating simulation-based learning into medical education. By addressing these issues, institutions can enhance the quality of medical education, improve clinical competency among students, and ultimately contribute to better patient care outcomes.

Recent research has identified that training using simulations serves a significant purpose of connecting educational theory with clinical competency transfer, providing an opportunity for skill standardization and professional preparation development (7–9).

However, there is a gap in strategic and systematic models regarding the construction and management of Medical Simulation Centers in the context of New Medical Sciences in China.

Thus, this paper contributes a grounded optimized institutional model based on Fujian Medical University’s experience to incorporate an enterprise of infrastructure, faculty policy, and curricular innovation into improving the sustainability and quality of Medical Simulation Centers.

## 2 Materials and methods

This research utilized a descriptive institutional design grounded in the practical experiences of Fujian Medical University’s Medical Simulation Center, which is envisioned to become a model under the New Medical Science framework. The research aimed to explore the processes, approaches, and challenges to developing and improving a modern Medical Simulation Center. This study investigated and contributed to developing and improving a sustainable simulation-based education center by examining the structural, management, and learning perspectives that underpin a Medical Simulation Center and whether a combination of infrastructure, faculty development, and curricular alignment improves the quality of medical education (10, 11).

### 2.1 Study settings and data sources

The study setting and data sources were developed based on institutional documents of Fujian Medical University maintained through official means for the years 2020–2024. Data sources included institutional teaching and training reports for each academic year, documentation of simulation session reports, faculty development documentation and records, and construction and management documentation of the Medical Simulation Center. Additional supporting documentation was also acquired, which included quality-assurance reports, documentary assessments of student assessments and performance, and assessments of clinical skills. These documents provided a rich source of institutional data for exploring the development, intent, and outcomes of research-based on the Medical Simulation Center in medical education (12–14).

### 2.2 Analysis process

A qualitative narrative synthesis was used for analysis of the institution documents. The narrative synthesis was necessary as it allows for the triangulation of quantitative indicators (e.g., number of simulations or hours of faculty training) with qualitative indicators (e.g., commentaries, policy, reports, or evaluations) into terms. The narrative synthesis intended to elicit significant themes and effective practices relating to infrastructure development, faculty capacity building, curricular alignment, and evaluation systems. The outcomes of thematic analysis of documents are reconstructed in Table 1, with recommendations related to key aspects and educational value of the research Medical Simulation Center that emerged from the study. Using iterative reading techniques and clumping techniques upon themes, effective practices gleaned from the Fujian Medical University model effectively practices, and participating concepts of consensus and widely held international commentary on a framework for medical education, effective practice (15, 16).

**Table 1:** Importance of Medical Simulation Centers.

Key aspects	Description
Bridging the gap between theory and practice	Provides an immersive and standardized learning experience to help students transition from theoretical knowledge to hands-on practice (7).
Enhancing procedural skills	Enables students to practice essential clinical procedures in a controlled environment, improving skill acquisition and retention (9).
Supporting competency-based medical education (CBME)	Aligns with CBME by emphasizing structured assessments, skill development, and measurable clinical competencies (2).
Encouraging interprofessional education	Allows medical, nursing, and allied health students to collaborate in team-based exercises, fostering teamwork and communication (10).
Improving clinical outcomes	Leads to better patient safety, reduced medical errors, and improved decision-making in real-world clinical settings (11).

### 2.3 Conceptual and theoretical framework

The analysis framework provided in the study drew from previous work undertaken as part of the study of Saratila et al. (17) and Romancenco et al. (33); the work found refers to the notion of simulation-based education being an opportunity to enhance the transfer of clinical competencies and to bring together theory-based content and practical applications. These frameworks were claimed and conceptually located in Competency-Based Medical Education (CBME), which outlines measurable outcomes, structured assessment, and the progressive nature of skill acquisition and assessment. The analytical process made sense of institutional reports and commentary into four categories of infrastructure and resources, faculty training and professional development, curricular alignments, and quality assurance networks. The analysis phenomena adhered to the original purpose of current best practices, while ensuring a sound conceptual analysis of the study within CBME (18–22).

### 2.4 Ethical considerations

No human or animal subjects participate in this study. All data were harvested from research-based documents held at Fujian Medical University, which did not compromise the confidentiality of participating human subjects, nor did the documents constitute any identifiable information about individuals used as data sources. This study did not contrive formal ethics committee recommendations; consequently, all facets of the process of data collection and analysis adhered to policies of the Fujian Medical University and Chinese national guidelines for transparency in educational research and confidentiality of research projects (23, 24).

## 3 Results

### 3.1 Achievements of the Medical Simulation Center

Between 2020 and 2024, the Medical Simulation Center at Fujian Medical University reached several significant milestones to advance simulation-based education. During this time, the number of simulation-based training labs increased from 8 to 14 in key areas, including emergency medicine, surgical skills, obstetrics and gynecology, and nursing. Regarding utilization, the increase in student participation grew from about 1,200 to more than 2,800 students per year, a growth of 133%. Additionally, the summers of 2023 include a successful institutional accreditation from the Fujian Provincial Department of Education, which recognized it as a program of best practices using competency-based medical education. In the summer of 2024, the center coordinated formal agreements with 2 regional teaching hospitals to begin to standardize simulation curriculum for clinical students to ensure equitable exposure (10–12).

### 3.2 Outcomes of training and student feedback

The systematic implementation of simulation training has resulted in measurable increases in student performance and satisfaction. Institutional data indicated that students who participated in at least three modules improved Objective Structured Clinical Examination (OSCE) scores between 15 and 20% above baseline scores (13, 14). Faculty also commented on an observable increase in accuracy when completing procedures, and also noted a reduction in demonstrating common technical errors during clinical placements. In more depth, for example, student satisfaction surveys (*n* = 650) collected in the summer of 2023–2024 indicated 94.3% of the students agreed that simulation helped students feel more confident in their skills, and 89.6% of students improved in timing and team communication after simulation training. After the internal “Simulation Instructor Development Program” was implemented, faculty involvement increased by 45% demonstrating greater institutional capacity (15, 16, 18).

### 3.3 Technology and innovations

To increase realism and learner engagement, the Medical Simulation Center has added digital tools that utilize the latest technology. This includes high-fidelity manikins for critical care training, virtual-reality (VR)-assisted surgical modules, and AI-assisted learning performance tracking systems. In addition to the educational residency program, more than one-third of simulation sessions involved the use of VR and AI by 2024, which adhered to worldwide standards for adaptive learning and learners’ personal skills assessment (19, 20). Student comments confirmed that technology was effective in enhancing engagement and scenario recall by over 30 % compared to non-electronic learning experiences.

### 3.4 Accreditation and quality assurance

To ensure consistency in evaluations of students, the institution adopted standardized evaluation tools, including OSCEs, Direct Observation of Procedural Skills (DOPS), and structured debriefing protocols. Accreditation requirements were aligned with standards established by the Society for Simulation in Healthcare (SSH) and the International Nursing Association for Clinical Simulation and Learning (INACSL) to ensure a worldwide perspective and authority of credibility.

Between the years of 2021 through 2024, the institution performed annual internal audits, and over 90% compliance was achieved to establish safety and scenario integrity, as well as learning evaluations (21–23).

### 3.5 Interprofessional and collaborative training

Interprofessional education (IPE) was achieved, and all curricular elements embrace collaborative activities where medical students and the school of nursing and pharmacy, and rehabilitation science education programs are represented. From 2022, the institution implemented team-based simulation sessions focused on scenarios of emergency response, perioperative collaboration, and patient communication.

Survey data demonstrated that 92% of respondents reported increased interprofessional understanding, and team performance during simulation multi-role scores increased by, on average, 18%. All evidence suggested that the Model was capable of strengthening teamwork and reducing team variability across professions (24–26).

### 3.6 Scalability and sustainability

To better ensure continued sustainability, in 2024, the center established a “shared-resource consortium” with three partnered teaching institutions providing risk-shared access to the simulator and educator resources. This consortium decreased the relative costs of simulator learning by approximately 28% per student while increasing accessibility of simulation learning experiences in rural teaching sites.

The educators also established the “Train-the-Trainer” program with the primary purpose of expanding the number of qualified instructors from 15 to 29 certified simulation educators within 2 years. As a result of the shared-resource consortium and the train-the-train program, the simulation program was established to promote ongoing maintenance and expansion for both education and clinical contexts.

The combination of long-term budget and financial planning and agreements to share resources elevates the Fujian Medical University Medical Simulation Center into a scalable and replicable model for medical education reform.

### 3.7 Medical Simulation Center achievements

The center successfully obtained institutional accreditation from the Fujian Provincial Department of Education in 2023, recognizing it as a model for competency-based medical education. In 2024, the center-initiated a collaboration with two regional teaching hospitals to standardize simulation curricula, ensuring consistent exposure for clinical students (10–12). A summary of key institutional outcomes from 2020 to 2024 is presented in Table 2.

**Table 2:** Summary of key outcomes from the Fujian Medical University Medical Simulation Center (2020–2024).

Indicator	Outcome	Supporting evidence
Institutional accreditation	Achieved Provincial Accreditation (2023)	Fujian dept. of education reports
Student participation	Increased from 1,200 → 2,800 annually (+133%)	Institutional records
OSCE performance	+15–20% score improvement post-training	Academic evaluation data
Faculty participation	+45% after the instructor program	Training logs
Student satisfaction	94.3% reported improved confidence	Survey 2023–2024
Technology adoption	35% sessions with VR/AI modules	Medical Simulation Center annual reports
Team-based simulation	92% improved interdisciplinary understanding	IPE feedback survey
Cost reduction	28% decrease in per-student training cost	Consortium financial data

## 4 Discussion

This article provides a detailed descriptive evaluation of Fujian Medical University’s Medical Simulation Center as a case of a simulation-based learning model incorporated into medical education through the New Medical Sciences perspective. The results illustrated the effectiveness of leveraging investment in infrastructure, faculty development, and curriculum innovation to enhance learner engagement and program sustainability.

### 4.1 Comparing with the literature

This study’s findings correlate well with the current literature available on simulation-based medical education. Saratila et al. (17) discussed that simulation-based learning results in significantly improved skill transfer and decision-making, which absolutely correlates with improvements in performance on the OSCE and with the student satisfaction measured in this study. Romancenco et al. (33) also highlighted faculty training and standardizing training scenarios as key ingredients to the quality of simulation programs, which was observed in this study as the model made incredible approaches through the “Simulation Instructor Development Program.” Furthermore, the reference to sustainability and interprofessional learning expands the work of Romancenco et al. (33), who suggested inter-institutional partnerships and shared resources to promote efficiency and sustainability for longer periods. By introducing a consortium framework, the model demonstrated how Fujian Medical University is utilizing partnerships in the region to maximize resources while taking care to maintain the quality of training (24–26).

On the other hand, the current study distinctly discusses the institutional management aspects, or administration, in utilizing alignment and ongoing quality checks to strengthen the effectiveness of learning.5.2 Implications for Institutions.

The Fujian model offers a replicable approach to Medical Simulation Centers, especially for medical schools in underdeveloped or resource-limited environments. Its integrated framework—across infrastructure, faculty, and curriculum—represents a well-balanced model for the institution that seeks to adopt competency-based medical education (CBME).

The findings demonstrate that ongoing faculty development, structured evaluation, and interprofessional training are all important to creating practice-ready outcomes for graduates. Evaluation systems that integrated accreditation required assessments suggested a shift from simulation being just a form of teaching, but now it has developed into the institution’s strategic asset.

Additionally, achieved 28% cost savings of the consortium model and generated enough certified educators, suggesting that institutional growth is sustainable without sacrificing educational quality, and provides a scale model (18, 27).

### 4.2 Limitations

There are limitations to this study despite its strengths. It was a single-center descriptive study, rather than multi-center comparative work, reliant on reports at the institution rather than comparative multi-center data. Therefore, while the findings give insight into structural and pedagogical effectiveness, the findings apply to the local context and need to be interpreted with caution.

The study lacked a longitudinal cohort for validating outcome measures for extending outcomes from simulation training into clinical practice.

Finally, the data in this study were primarily descriptive; theoretical performance was limited to fractionating and statistical summarizing, and future research aims should incorporate mixed methods or quasi-experimental designs to clarify or develop stronger causal links (17, 28, 29).

### 4.3 Future directions

Novel technologies, such as virtual reality (VR) or artificial intelligence (AI), and tele-simulation, are changing medical education in a global context. Implementation of these modalities can support educational experiences by enhancing realism, differentially adapting training levels while maintaining forward progress, and extending access to remotely located learners.

In a Fujian context, implementation of performance analytics could further individualize simulation learning based on overall performance trends, and tele-simulation could create space for inter-campus center training across entities (7, 30–32).

In the future, as education evolves, simulation-based learning will establish a back-and-forth relationship between technological technology and pedagogical integrity. Institutions will still need to be learner-focused, making sure that simulation is a complement—not a substitute for clinical experience.

## 5 Conclusion

The establishment of a well-organized, faculty-led Medical Simulation Center enhances the outcomes of competency-based medical education. The experience from Fujian Medical University demonstrates that systematic faculty management, continuous professional development, and curricular integration are key strategies for achieving sustainable simulation-based training. This model provides a practical reference for institutions seeking to modernize medical education and strengthen clinical competence among learners.

Collaboration should continue to expand across multiple universities and disciplines, accompanied by the integration of innovative technologies such as virtual reality, artificial intelligence, and tele-simulation to further advance educational reform.

Future studies should evaluate the long-term impact of simulation-based training on graduates’ clinical competence and patient outcomes. Comparative and longitudinal analyses across institutions would help determine whether simulation exposure translates into improved clinical performance, safety practices, and sustained professional development. Such evidence will be critical for establishing national standards and policy frameworks that support competency-based medical education.

## Data Availability

The raw data supporting the conclusions of this article will be made available by the authors, without undue reservation.
